# 

**Published:** 1882-03

**Authors:** 


### CONTENTS:

PAGE.

ORIGINAL COMMUNICATIONS:

Variola in Buffalo. By J. I. Marcley, M. D., 337

CLINICAL REPORTS:

Foreign Bodies in the Intestines. Reported by Thomas Lothrop, M. D., . . . . 346
Report of Two Cases of Ovariotomy Successfully Performed. By Matthew D. Mann, A. M., M. D., . . . . . 351

SELECTIONS:

The Treatment of Syphilis by Subcutaneous Injection. . . . . . . . . . 356
The Pathology of Malaria, . . . . . 362
The Debate on Salicin and the Salicylates in Rheumatism, . . . . . . . . 365
Is "Congestion of the Brain" a Correct Pathological Expression? . . . 367
Tabes and Syphilis, . . . . . . 370
The Characteristic Appearance of Wounds of the Intestines Made During Life, . 371
E. Pasteur's Experiments on Charbon, . 372
Diabetes Mellitus and Albuminuria, . . 373
Vaso-Motors of the Lymphatics, . . . 374
Writer's Cramp, . . . . . . . . 374
Alterations of the Nerves in Chronic Rheumatism, . . . . . . . . 374
Constipation in Infants, . . . . . 375

EDITORIAL:

The Board of Regents and Medical Colleges, 375
The Code of Ethics, . . . . . . 377
Society of the Red Cross, . . . . . 378
Council of the University of Buffalo, . . 379
Buffalo Medical College—Annual Commencement, . . . . . . . . . . 379

EDITORIAL NOTES, . . . . . 381

REVIEWS, . . . . . . . 384

